# Adipocyte YTH N(6)-methyladenosine RNA-binding protein 1 protects against obesity by promoting white adipose tissue beiging in male mice

**DOI:** 10.1038/s41467-023-37100-z

**Published:** 2023-03-13

**Authors:** Sujun Yan, Xiaoling Zhou, Canlan Wu, Yunyi Gao, Yu Qian, Jingyu Hou, Renxiang Xie, Bing Han, Zhanghui Chen, Saisai Wei, Xiangwei Gao

**Affiliations:** 1 Sir Run-Run Shaw Hospital, Institute of Environmental Medicine, Zhejiang University School of Medicine, Hangzhou, China; 2 Department of Clinical Laboratory, Sir Run Run Shaw Hospital, Zhejiang University School of Medicine, Hangzhou, China; 3 Zhanjiang Institute of Clinical Medicine, Zhanjiang Central Hospital, Guangdong Medical University, Zhanjiang, China; 4 Key Laboratory of Laparoscopic Technology of Zhejiang Province, Department of General Surgery, Sir Run-Run Shaw Hospital, Zhejiang University School of Medicine, Hangzhou, China; 5 Bioelectromagnetics Laboratory, Zhejiang University School of Medicine, Hangzhou, China

**Keywords:** RNA modification, Fat metabolism, Obesity

## Abstract

Obesity, one of the most serious public health issues, is caused by the imbalance of energy intake and energy expenditure. N(6)-methyladenosine (m^6^A) RNA modification has been recently identified as a key regulator of obesity, while the detailed mechanism is elusive. Here, we find that YTH RNA binding protein 1 (YTHDF1), an m^6^A reader, acts as an essential regulator of white adipose tissue metabolism. The expression of YTHDF1 decreases in adipose tissue of male mice fed a high-fat diet. Adipocyte-specific *Ythdf1* deficiency exacerbates obesity-induced metabolic defects and inhibits beiging of inguinal white adipose tissue (iWAT) in male mice. By contrast, male mice with WAT-specific YTHDF1 overexpression are resistant to obesity and shows promotion of beiging. Mechanistically, YTHDF1 regulates the translation of diverse m^6^A-modified mRNAs. In particular, YTHDF1 facilitates the translation of bone morphogenetic protein 8b (*Bmp8b*) in an m^6^A-dependent manner to induce the beiging process. Here, we show that YTHDF1 may be an potential therapeutic target for the management of obesity-associated diseases.

## Abstract (web summary)

Activation of white adipose tissue (WAT) thermogenesis alleviates obesity-associated metabolic disorders in rodents. Here the authors report that the m6 A RNA modification reader YTHDF1 promotes WAT thermogenesis in a study with male mice, and may be a potential target for the treatment of obesity.

## Introduction

Adipose tissue is traditionally categorized into white adipose tissue (WAT) and brown adipose tissue (BAT), depending on its morphology and function^1^. WAT possesses unilocular, large lipid droplets and is involved in lipid storage. Excessive lipid storage in WAT results in obesity and related metabolic disorders, including insulin resistance, hepatic steatosis, diabetes, and cardiovascular disease^2,3^. By contrast, BAT possesses multilocular, small lipid droplets and a large number of mitochondria, in which uncoupling protein 1 (UCP1) is expressed, specializing in energy production in the form of heat^4^. Emerging evidence has identified a third type of adipose tissue, termed “beige” adipose tissue, which can be induced from WAT in cases of cold exposure, exercise, diet, and various activators^5,6^. Beige adipose tissue has unique origins and molecular characteristics compared with classic BAT^7^. Activation of thermogenesis in brown or beige adipose tissues increases systemic energy expenditure and alleviates obesity-associated metabolic diseases^8,9^. Thus, a deeper understanding of the mechanisms regulating energy storage and expenditure may lead to the development of therapeutic strategies that improve metabolic health.

N(6)-methyladenosine (m^6^A), the most abundant internal modification on mRNA, is catalyzed by the RNA methyltransferase complex methyltransferase-like (METTL) 3/METTL14/WT1-associated protein^10–12^. As a reversable modification, m^6^A methylation can be removed by AlkB homolog 5 or fat mass and obesity-associated protein (FTO)^13,14^. The YTH domain-containing ‘reader’ proteins, bind m^6^A and differentially regulate mRNA metabolism, including mRNA splicing, maturation, degradation, and translation. YTHDF1 enhances the translation of m^6^A-modified mRNAs, while YTHDF2 promotes the degradation of m^6^A-modified mRNAs^15,16^. YTHDF3 was reported to regulate both mRNA translation and degradation^17^.

Recent studies have highlighted an essential role of m^6^A modification in adipocytes metabolism. Single-nucleotide polymorphisms in *FTO* are closely related to the occurrence of obesity^18,19^, and knockout of *Fto* decreases HFD-induced obesity^20^. Coordinately, FTO inhibitors can increase thermogenesis, improve glucose tolerance, and ultimately inhibit obesity^21^. Moreover, METTL3 is essential for controlling postnatal development and energy homeostasis in BAT^22^. Because the fates of methylated mRNAs, ranging from degradation to translation, are determined by their reader proteins^15,16,23,24^, deciphering the functions of m^6^A reader proteins may help elucidate the mechanisms of m^6^A function in adipocyte metabolism.

In this work, we assessed the role of YTHDF1 in adipose tissue using WAT-specific *Ythdf1*-knockout male mice and adeno-associated virus^25^-mediated *Ythdf1* overexpression. We demonstrated that YTHDF1 promotes mRNA translation to induce WAT beiging and alleviate obesity. Our results highlighted an important role of YTHDF1 in preventing obesity and provided potential targets for the treatment of obesity-associated metabolic diseases.

## Results

### Adipose YTHDF1 expression is reduced in obesity

**Fig. 1:**
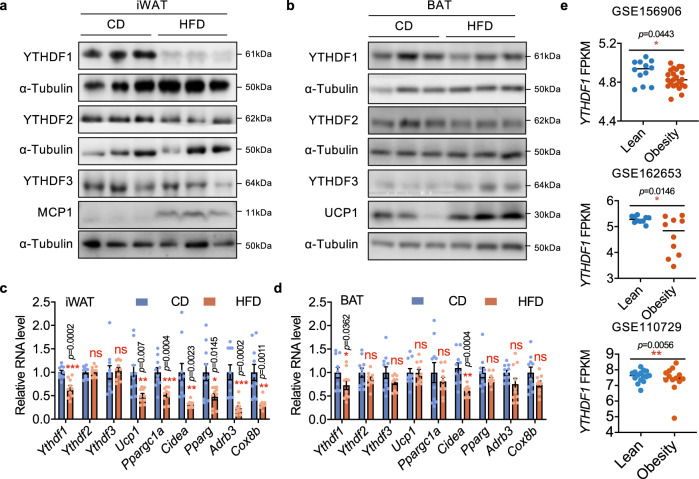
Adipose YTHDF1 expression is reduced in obesity. **a**, **b** Immunoblot analysis of YTH family proteins in iWAT **a** and BAT **b** of mice fed with CD or HFD for 12 weeks. **c, d** mRNA levels of *Yth* family genes and thermogenesis-related genes in iWAT **c** and BAT **d** of CD- and HFD-fed mice. Data were presented as mean ± SEM (*n* = 10). ns, not significant, *P < 0.05, **P < 0.01, ***P < 0.001, two-sided t-test. **e** Expression of *YTHDF1* in human white adipose tissues from GSE156906 (*n*_Lean_ = 14, *n*_Obesity_ = 27, two-sided t-test), GSE162653 (*n* = 10, two-sided t-test), and GSE110729 (*n*_Lean_ = 15, *n*_Obesity_ = 13, F-test). Source data are provided as a Source Data file.

Dysregulated m^6^A modification results in obesity in both mice and human beings^18,19^. To investigate the functions of “reader” proteins in this process, we detected the expression of YTH proteins (YTHDF1/2/3) in mice fed a standard chow diet (CD) or obese mice induced by a high fat diet (HFD). We observed a dramatic increase in body weight, iWAT weight, eWAT weight and BAT weight, indicating the successful induction of obesity (Supplementary Fig. 1A). Interestingly, YTHDF1 was dramatically downregulated in inguinal WAT (iWAT) in obese mice compared with that in control mice (Fig. 1a, c, Supplementary Fig. 1B, C). The expression of YTHDF2/3 did not change in both iWAT and BAT of HFD-induced mice (Fig. 1a–d, Supplementary Fig. 1B–E). Notably, HFD-fed mice exhibited lower expression of thermogenic genes (e.g., *Ucp1*, *Ppargc1a*, *Cidea*, *Pparg*, *Adrb3*, and *Cox8b*) in iWAT, implying impaired thermogenesis (Fig. 1c). Importantly, the published sequencing data revealed decreased *YTHDF1* mRNA expression in WAT of individuals with obesity compared with that in nonobese individuals (Fig. 1e). The negative correlation of YTHDF1 expression in WAT with obesity implies a role of this protein in adipose tissue metabolism.

### Adipocyte-specific knockout of *Ythdf1* predisposes mice to HFD-induced obesity

**Fig. 2:**
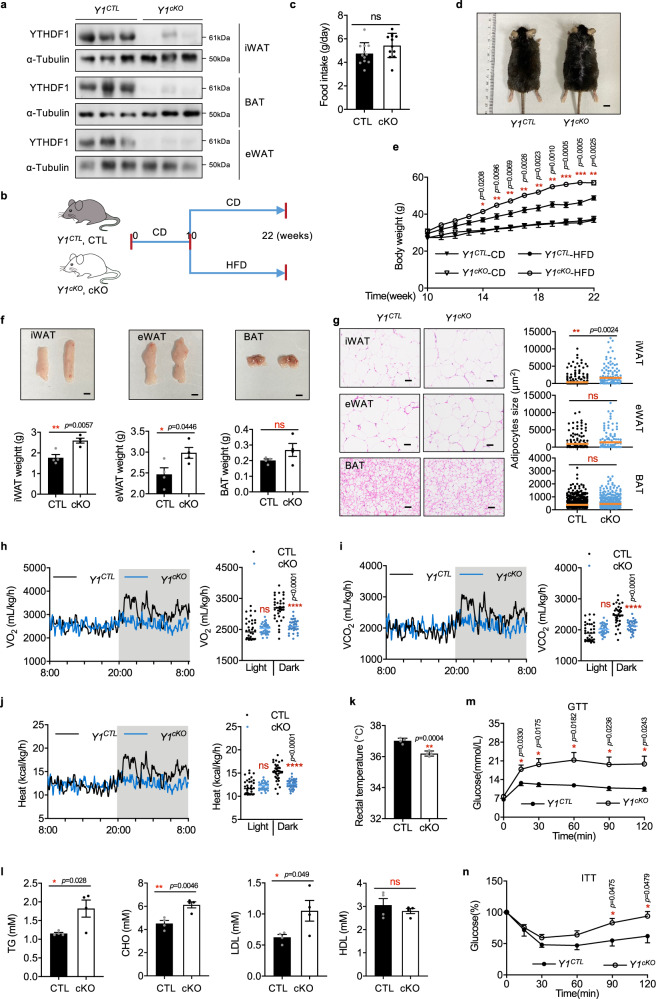
Adipocyte-specific knockout of *Ythdf1* predisposes mice to HFD-induced obesity. **a** Immunoblotting of adipose tissues from *Y1*^*CTL*^ and *Y1*^*cKO*^ mice. **b** Schematic of the mouse treatment regimen. **c**–**n** The *Y1*^*CTL*^ and *Y1*^*cKO*^ littermates were fed with HFD. **c** Daily food intake by *Y1*^*CTL*^ and *Y1*^*cKO*^ mice. **d** Gross view of *Y1*^*CTL*^ and *Y1*^*cKO*^ mice. Scale bar, 0.5 cm. **e** Body weights of *Y1*^*CTL*^ and *Y1*^*cKO*^ mice. **f** Gross view and weights of iWAT, eWAT, and BAT from *Y1*^*CTL*^ and *Y1*^*cKO*^ mice. Scale bar, 0.5 cm. **g** H&E staining of iWAT eWAT, and BAT from *Y1*^*CTL*^ and *Y1*^*cKO*^ mice. Scale bar, 50 μm. The red line indicated the average size. No adjustments. **h**–**j** O_2_ consumption **h**, CO_2_ generation **i**, and energy heat generation **j** of *Y1*^*CTL*^ and *Y1*^*cKO*^ mice. White and gray areas in the graphs indicate day and night, respectively. **k** Rectal temperature in *Y1*^*CTL*^ and *Y1*^*cKO*^ mice. **l** Serum concentrations of TG, CHO, LDL, and HDL in *Y1*^*CTL*^ and *Y1*^*cKO*^ mice fed with HFD. **m**, **n** glucose tolerance **m** and Insulin tolerance **n** fed with HFD. Data in **c**, **e**–**n** were presented as mean ± SEM (*n* = 4 biologically independent mice). ns, not significant, *P < 0.05, **P < 0.01, ****P < 0.0001, two-sided t-test. Source data are provided as a Source Data file.

To study the function of YTHDF1 in adipose tissue, we generated adipocyte-conditional *Ythdf1*-knockout mice (*Ythdf1*^*cKO*^ mice, termed *Y1*^*cKO*^ or cKO), which were produced by crossing *Ythdf1*^*flox/flox*^ mice (*Ythdf1*^*CTL*^ mice, termed *Y1*^*CTL*^ or CTL) with Adipoq-Cre transgenic mice (Supplementary Fig. 2A, Fig. 2a). Knockout of *Ythdf1* did not affect the expression of YTHDF2/3 in iWAT and BAT (Supplementary Fig. 2B, C). CD-fed *Ythdf1*^*cKO*^ mice and *Ythdf1*^*CTL*^ littermates showed similar weight gain and similar iWAT, BAT, and eWAT weights (Supplementary Fig. 2D, E). Consistent with these results, there were no difference in triglyceride (TG), total cholesterol (CHO), or low-density lipoprotein (LDL) levels between *Ythdf1*^*CTL*^ and *Ythdf1*^*cKO*^ mice after CD feeding (Supplementary Fig. 2F). Analyses of intraperitoneal glucose tolerance and insulin resistance, as measured using glucose tolerance tests (GTTs) and insulin tolerance tests^26^, showed no significant difference between CD-fed *Ythdf1*^*CTL*^ and *Ythdf1*^*cKO*^ mice (Supplementary Fig. 2G, H).

To clarify the function of YTHDF1 in obesity, *Ythdf1*^*CTL*^ and *Ythdf1*^*cKO*^ mice were fed an HFD starting at 10 weeks of age to induce obesity (Fig. 2b). Although CD-fed *Ythdf1*^*cKO*^ mice and *Ythdf1*^*CTL*^ littermates showed similar weight gain (Fig. 2e), HFD-fed *Ythdf1*^*cKO*^ mice showed significantly higher body weights compared with *Ythdf1*^*CTL*^ littermates, despite comparable food intake (Fig. 2c–e). Examination of dissected fat tissues confirmed that epididymal and inguinal but not brown fat pads were larger in *Ythdf1*^*cKO*^ mice (Fig. 2f). Moreover, hematoxylin and eosin (H&E) analysis revealed that the WAT in *Ythdf1*^*cKO*^ mice showed enhancement of obesity-related features, including larger unilocular lipid droplets (Fig. 2g). Taken together, these data suggested that *Ythdf1*^*cKO*^ mice were more prone to HFD-induced obesity.

### Adipocyte-specific knockout of *Ythdf1* aggravates obesity-induced metabolic disorders

The observed increase in body weight after HFD feeding, independent of food intake, suggested increased energy storage and reduced thermogenesis by *Ythdf1*^*cKO*^ mice. Indeed, metabolic cage experiments demonstrated that deletion of *Ythdf1* inhibited oxygen consumption, carbon dioxide generation, and energy heat generation during dark cycles (Fig. 2h–j). The core temperature was significantly reduced in *Ythdf1*^*cKO*^ mice (Fig. 2k). HFD feeding can elevate circulating levels of TG, CHO, and LDL^27^. We observed a further increase in these parameters in HFD-fed *Ythdf1*^*cKO*^ mice (Fig. 2l). Notably, GTTs and ITTs showed that *Ythdf1*^*cKO*^ mice fed an HFD developed glucose intolerance and insulin resistance (Fig. 2m, n). Collectively, our data demonstrated that adipocyte-specific knockout of *Ythdf1* aggravated the detrimental effects of obesity.

Furthermore, we fed the *Ythdf1*^*CTL*^ and *Ytdhf1*^*cKO*^ mice HFD under thermoneutral conditions. Deletion of *Ythdf1* exacerbated obesity, reduced rectal temperature, and downregulated the thermogenic genes (Supplementary Fig. 3A–E). Metabolic cage experiments showed that deletion of *Ythdf1* inhibited oxygen consumption, carbon dioxide generation, and energy heat generation during both light and dark cycles (Supplementary Fig. 3F–H). These data implied that the metabolic changes in *Ythdf1*^*cKO*^ mice might be caused by impaired thermogenesis of iWAT.

### Adipocyte-specific knockout of *Ythdf1* inhibits the beiging of WAT

**Fig. 3:**
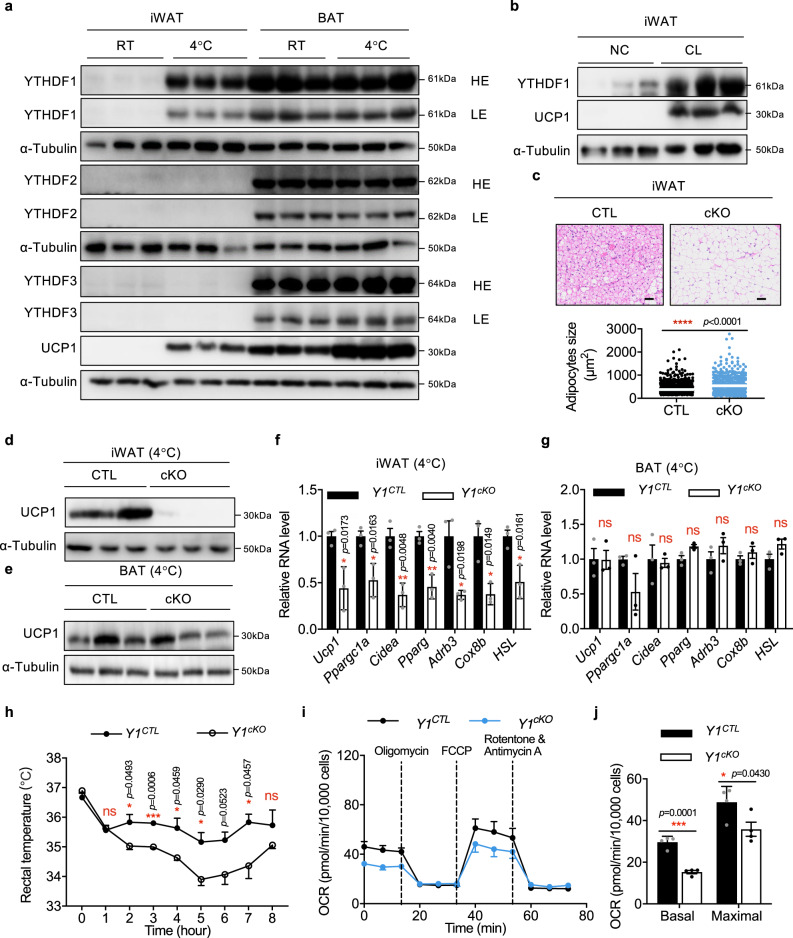
Adipocyte-specific knockout of *Ythdf1* inhibits beiging of iWAT. **a** Immunoblot analysis of YTH family proteins expression in iWAT and BAT from mice housed at room temperature (RT) or 4 °C. **b** Immunoblot analysis of YTHDF1 and UCP1 expression in iWAT from mice treated with or without CL-316,243 (CL). **c**–**g** *Y1*^*CTL*^ and *Y1*^*cKO*^ littermates were exposed to cold conditions for 7 days. **c** H&E staining of iWAT. Scale bar, 50 μm. The white line indicated the average size. No adjustments. **d**, **e** Immunoblot analysis of UCP1 in iWAT **d** and BAT **e**. **f**, **g** mRNA levels of thermogenesis- and lipolysis-related genes in iWAT (**f**) and BAT (**g**) Data in **f, g** were presented as mean ± SEM (*n* = 3 biologically indipendent mice). No adjustments. **h** Rectal temperature measured after exposure to cold conditions for up to 8 h, as indicated. Data were presented as mean ± SEM (*n* = 3 biologically independent mice). **i** The O_2_ consumption rate (OCR) in primary preadipocytes isolated from *Y1*^*CTL*^ and *Y1*^*cKO*^ mice determined by Seahorse. **j** The average basal and maximal respiration rates. Data in **i**, **j** were presented as mean ± SEM **(***n* = 10,000 cells exmined over 3 independent experiments). ns, not significant, *P < 0.05, **P < 0.01, ***P < 0.001. All of the *P*-values are determined by unpaired two-sided t-test. Source data are provided as a Source Data file.

Studies have demonstrated that beige adipose tissue plays active roles in lipid metabolism and has potential therapeutic relevance for weight loss^8,28^. Beiging is induced by chronic exposure to external cues, such as cold treatment or adrenergic stimulation^5,6^. To investigate whether YTHDF1 modulates the functions of beige adipose tissues, we induced the beiging process. YTHDF1, but not YTHDF2/3, was upregulated in cold-treated iWAT (Fig. 3a and Supplementary Fig. 4A, B). β3-Adrenergic receptor (AR) is the main signaling protein involved in the beiging process. Intraperitoneal administration of the β3-AR agonist CL-316,243 (CL) resulted in the upregulation of YTHDF1 (Fig. 3b). Histological and immunohistochemical analyses showed that the lipid droplets were larger in *Ythdf1*^*cKO*^ mice than that in *Ythdf1*^*CTL*^ mice after cold stimulation (Fig. 3c). UCP1 expression was also dramatically downregulated in iWAT of *Ythdf1*^*cKO*^ mice (Fig. 3d). Overexpression of YTHDF2/3 could not rescue the phenotype of *Ythdf1*^*cKO*^ (Supplementary Fig. 4C, D), implying differential roles of YTHDF1/2/3 in adipose tissue. Interestingly, UCP1 in BAT was unchanged in *Ythdf1*^*cKO*^ mice after cold stimulation (Fig. 3e). Other thermogenesis- and lipolysis-related genes were both downregulated in iWAT rather than BAT (Fig. 3f, g and Supplementary Fig. 4E, F), suggesting that YTHDF1 may have a major role in WAT rather than BAT. Consistently, the rectal temperatures of *Ythdf1*^*cKO*^ mice remained lower after cold exposure (Fig. 3h). The primary preadipocytes were further isolated from *Ythdf1*^*CTL*^ and *Ythdf1*^*cKO*^ mice and the thermogenic program was determined. Deletion of *Ythdf1* dramatically reduced the O_2_ consumption rate (OCR) in primary preadipocytes (Fig. 3i).

To exclude BAT function, we surgically removed BAT. Without BAT, *Ythdf1*^*cKO*^ mice still had lower OCR and lower *Ucp1* expression than *Ythdf1*^*CTL*^ mice under RT, cold, and thermoneutral conditions (Supplementary Fig. 4G-I). Taken together, these data suggested that YTHDF1 promoted the thermogenesis of adipocytes in WAT.

### Transcriptome-wide identification of YTHDF1-regulated transcripts in beige adipose tissue

**Fig. 4:**
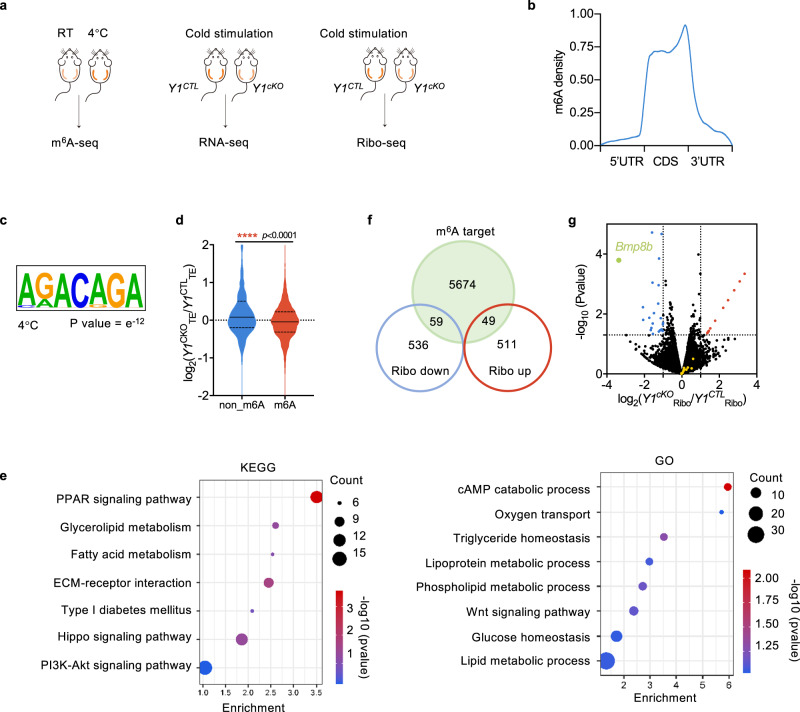
Transcriptome‐wide identification of YTHDF1‐regulated transcripts in beige adipose tissue. **a** Schematic description of the m^6^A-seq and RNA-seq, and Ribo-seq experiments. **b** Metagene plot of m^6^A peak distribution in iWAT at 4 °C. **c** Consensus motif of m^6^A sites in beige adipose tissue. **d** Violin plots showing TE changes between *Ythdf1*^*CTL*^ and *Ythdf1*^*cKO*^ mice for nonmethylated (non‐m^6^A) and methylated (m^6^A) transcripts. ****P < 0.001, Mann–Whitney test. No adjustments. **e** Top KEGG and GO analysis terms enriched for transcripts during beiging and downregulated translation levels. **f** Overlap of m^6^A targeted transcripts with translationally downregulated or upregulated genes. **g** Volcano plot of fold changes in translation levels from iWAT of cold-treated *Y1*^*CTL*^ and *Y1*^*cKO*^ mice. The upregulated (red) and downregulated (blue) genes are highlighted. Green dot indicates the *Bmp8b* gene. The other BMP genes were highlighted in yellow. Mann–Whitney test. Source data are provided as a Source Data file.

Because YTHDF1 is an m^6^A reader protein, we performed m^6^A-seq in cold treated iWAT (Fig. 4a). As expected, our m^6^A data revealed that the distribution of m^6^A was enriched around stop codons within transcripts (Fig. 4b). The m^6^A peaks were characterized by the canonical RGACH motif (Fig. 4c). YTHDF1 is known to affect mRNA translation^15^. To identify potential targets regulated by YTHDF1, we assessed changes in mRNA levels and translational efficiency by RNA-seq and ribosome profiling (Ribo‐seq) in wild-type and *Ythdf1*-knockout iWAT under cold stimulation (Fig. 4a). As expected, our data revealed a notable decrease in translational efficiency for m^6^A‐marked transcripts in *Ythdf1*-knockout mice compared with that in wild-type mice (Fig. 4d).

To identify the functional pathways associated with YTHDF1‐targeted mRNAs, we analyzed genes that were translationally altered by *Ythdf1* knockout. In total, we detected 595 downregulated genes and 560 upregulated genes from Ribo-seq data. Kyoto Encyclopedia of Genes and Genomes pathways analyses showed that the top enriched pathways of downregulated genes were associated with a series of metabolism-related pathways such as PPAR signaling pathway and fatty acid metabolism. Gene Ontology analysis revealed that cAMP catabolic process, TG homeostasis, and lipoprotein metabolic process were enriched (Fig. 4e). The upregulated genes were enriched in nicotine addiction and cardiac muscle contraction (Supplementary Fig. 5A). By overlapping differentially expressed genes with m^6^A-modified mRNAs, we identified 59 downregulated genes and 49 upregulated genes with m^6^A modification, which are potential YTHDF1 targets. (Fig. 4f). Among the 59 genes, bone morphogenetic protein 8b (*Bmp8b*) was the most dramatically downregulated one (Fig. 4g). Therefore, we picked BMP8B for further validation.

### YTHDF1 regulates BMP8B in an m^6^A-dependent manner

**Fig. 5:**
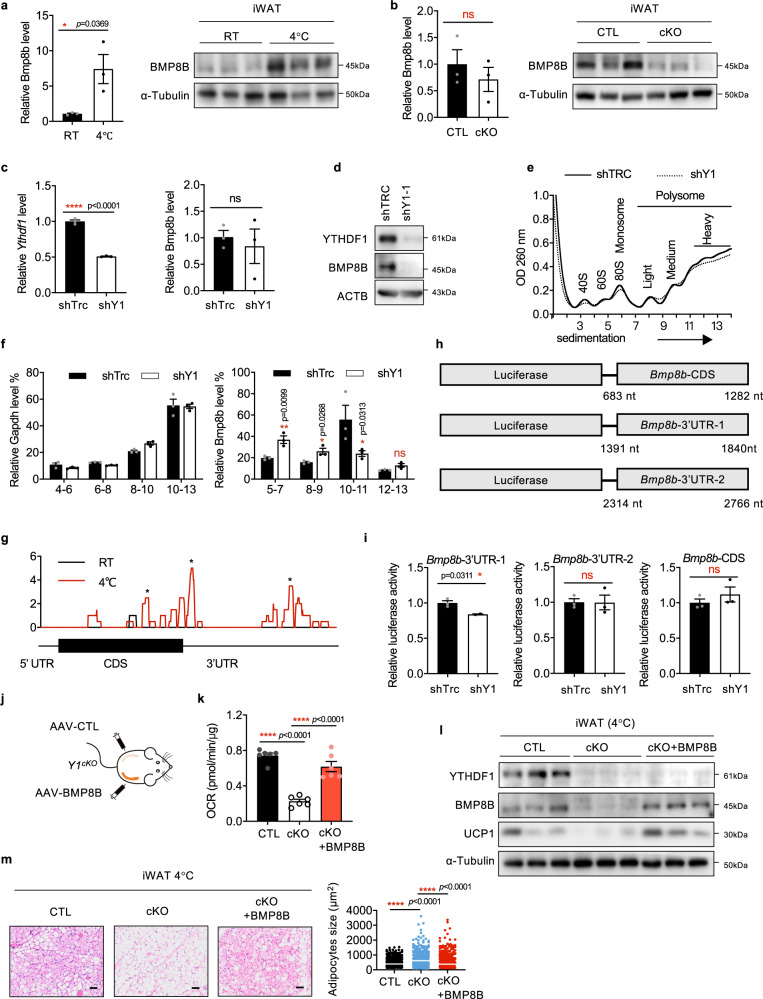
YTHDF1 regulates BMP8B in an m^6^A-dependent manner. **a** The mRNA and protein levels of BMP8B in iWAT from mice treated at RT or 4 °C. **b** The mRNA and protein levels of BMP8B in iWAT from *Y1*^*CTL*^ and *Y1*^*cKO*^ mice treated at 4 °C. **c** The mRNA level of *Ythdf1* and *Bmp8b* in 3T3-L1 cells with or without YTHDF1 knockdown. **d** The protein levels of BMP8B expression in 3T3-L1 cells with or without *Ythdf1* knockdown. **e** Polysome profiles of 3T3-L1 cells with or without YTHDF1 knockdown. **f** The distributions of *Gapdh* and *Bmp8b* in polysome fractions. **g** m^6^A peak distribution within *Bmp8b* transcripts. * indicates the predicted m^6^A peak. **h** Schematic of luciferase constructs with the predicted m^6^A sites in the CDS and 3′UTR of *Bmp8b* mRNA. **i** Luciferase activity of the *Bmp8b* reporter in 3T3-L1 cells with or without YTHDF1 knockdown. **j** Schematic of the mouse treatment regimen. **k–m** Mice were treated at 4 °C. **k** Oxygen consumption rates (OCR) of iWAT from *Y1*^*CTL*^ and *Y1*^*cKO*^ mice with or without BMP8B overexpression. Data were presented as mean ± SEM (*n* = 6 biologically independent mice). ****P < 0.0001. **l** The expression of BMP8B and UCP1 in iWAT from *Y1*^*CTL*^ and *Y1*^*cKO*^ mice with or without BMP8B expression. **m** H&E staining of iWAT depots from *Y1*^*CTL*^ and *Y1*^*cKO*^ mice with or without BMP8B expression. Scale bar, 50 μm. The white line indicated the average size. No adjustments. Data in **a–c, f**, and **i** were presented as mean ± SEM (*n* = 3). ns, not significant, *P < 0.05, ****P < 0.0001. All of the *P*-values are determined by unpaired two-sided t-test. Source data are provided as a Source Data file.

The elevated expression of BMP8B in beige adipose tissue was confirmed, implying a role of BMP8B in iWAT beiging (Fig. 5a). The *Bmp8b* mRNA was almost unchanged, while BMP8B protein level was downregulated in *Ythdf1*^*cKO*^ iWAT, implying that YTHDF1 regulated BMP8B expression at the translational level (Fig. 5b). We analyzed BMP8B expression in BAT from *Ythdf1*^*CTL*^ and *Ythdf1*^*cKO*^ mice. Knockout of *Ythdf1* did not affect BMP8B protein level and mRNA translation in BAT (Supplementary Fig. 5B–D), suggesting that YTHDF1 regulates *Bmp8b* translation mainly in iWAT.

To elucidate the regulatory mechanism through which YTHDF1 affected BMP8B expression, we generated *Ythdf1*-knockdown pre-adipocyte 3T3-L1 cells (Fig. 5c), which also exhibited decreased BMP8B protein level but no change in *Bmp8b* mRNA level (Fig. 5c, d). Polysome profiling revealed reduced translation of *Bmp8b* in *Ythdf1*‐silenced cells (Fig. 5e, f). Knockdown of YTHDF2 or YTHDF3 did not affect BMP8B expression (Supplementary Fig. 5E, F), implying that BMP8B expression is specifically regulated by YTHDF1. Three m^6^A peaks were identified in the coding sequence (CDS) and 3’ untranslated region (UTR) of *Bmp8b* mRNA (nt 683–1282, 1391–1840, and 2314–2766 in the transcript ENSMUSG00000002384; Fig. 5g). To investigate the regulation of BMP8B by YTHDF1 through m^6^A, we cloned each of the m^6^A peaks into a luciferase reporter (Fig. 5h). Knockdown of *Ythdf1* decreased *Bmp8b*−3’UTR-1 luciferase activity but not *Bmp8b*-CDS or *Bmp8b*−3’UTR-2 luciferase activity (Fig. 5i), suggesting that YTHDF1 mediated the translation of the *Bmp8b* 3’UTR. The binding of YTHDF1 with *Bmp8b* mRNA was confirmed by RIP-qPCR analysis (Supplementary Fig. 5G). Collectively, these findings suggested that the translation of *Bmp8b* was directly regulated by YTHDF1.

To clarify the functional relationship between BMP8B and YTHDF1, we performed rescue experiments by expressing FLAG-tagged BMP8B in *Ythdf1*-depleted iWAT using an AAV system (Fig. 5j). Overexpression of BMP8B largely reversed the impaired beiging, changes in UCP1 expression, and OCR elicited by *Ythdf1* deletion (Fig. 5k–m). These results indicated that BMP8B was a key effector promoting WAT beiging downstream of YTHDF1.

### WAT-specific YTHDF1 overexpression promotes beiging

**Fig. 6:**
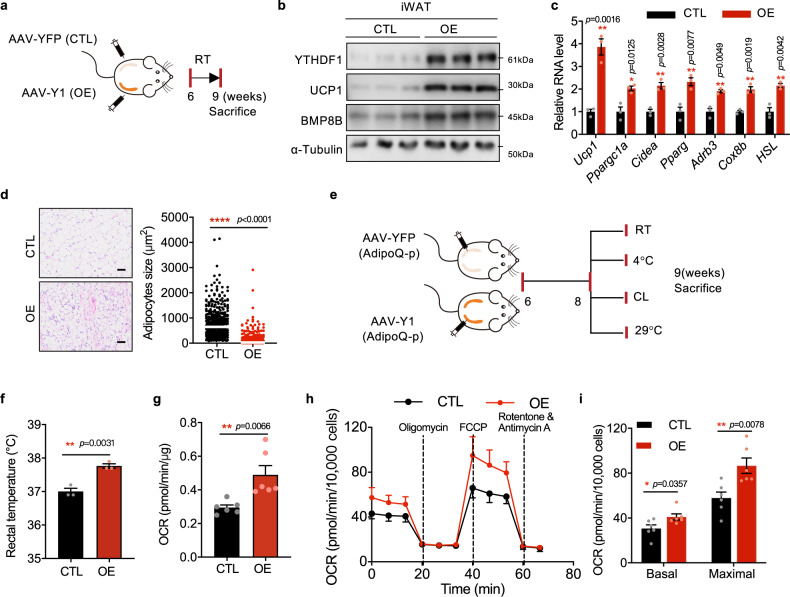
WAT-specific YTHDF1 overexpression promotes beiging. **a** Schematic of the mouse treatment regimen. **b** Immunoblot analysis of YTHDF1, UCP1 and BMP8B in iWAT. **c** mRNA levels of thermogenesis-related genes. Data were presented as mean ± SEM (*n* = 4). ns, not significant, *P < 0. 05. **d** H&E staining of iWAT. Scale bar, 50 μm. The white line indicated the average size. No adjustments. **e** Schematic of the mouse treatment regimen. **f** Rectal temperature of Y1^CTL^ and Y1^OE^ littermates. t-test. **g** The OCR of iWAT from CTL and OE mice. **f**, **g** Data were presented as mean ± SEM (*n* = 6). **P < 0.01, ***P < 0.001. **h** The OCR in primary preadipocytes isolated from CTL and OE mice determined by Seahorse. **i** The average basal and maximal respiration rates in primary preadipocytes. Data in **h, i** were presented as mean ± SEM (*n* = 10,000 cells exmined over 3 independent experiments). *P < 0. 05, **P < 0.01. All of the *P*-values are determined by unpaired two-sided t-test. Source data are provided as a Source Data file.

We hypothesized that WAT-specific YTHDF1 overexpression may promote the beiging process and ameliorate HFD-induced obesity. Direct unilateral injection of AAVs expressing YTHDF1 (Y1^OE^) or yellow fluorescent protein (YFP) (Y1^CTL^) into the inguinal fat pads of mice was performed. All mice in this cohort were housed in individual cages at room temperature to reduce variability in the individual degree of WAT beiging. Mice were sacrificed after 3 weeks of recovery from surgery (Fig. 6a). Our findings showed that the expression of UCP1 and BMP8B was evaluated in Y1^OE^ side than Y1^CTL^ side, with a dramatical increase in YTHDF1 overexpression (Fig. 6b). The mRNA levels of thermogenesis- and lipolysis-related genes were elevated (Fig. 6c). We observed an increase in iWAT beiging in Y1^OE^ fat pads (Fig. 6d). To achieve adipocyte-specific expression, we used adiponectin-promoter to drive YTHDF1 expression and got similar results (Supplementary Fig. 6A, B).

In another group, bilateral injection of YTHDF1- or YFP-AAV into the inguinal fat pads of mice was performed (Fig. 6e). The rectal temperature, OCR, *Ucp1* expression, and lipid droplets in iWAT of Y1^OE^ mice were dramatically increased under cold, RT, thermoneutral condition, and NE stimulation (Fig. 6f, g and Supplementary Fig. 6C–E), supporting the enhanced thermogenic capacity. The OCR of preadipocytes isolated from Y1^OE^ mice was dramatically increased (Fig. 6h). Overall, these findings indicated that overexpression of YTHDF1 in iWAT caused spontaneous WAT beiging and adipocyte thermogenesis.

### WAT-specific YTHDF1 overexpression ameliorates HFD-induced obesity

**Fig. 7:**
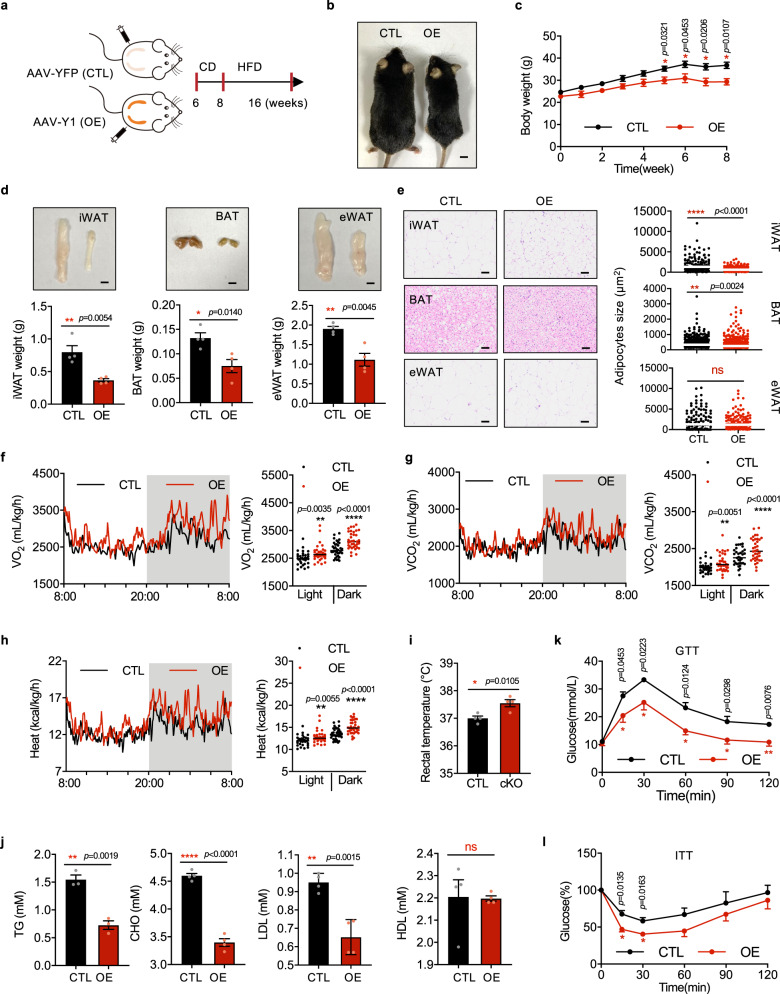
WAT-specific YTHDF1 overexpression ameliorates HFD-induced obesity. **a** Schematic of the mouse treatment regimen. **b**–**l** The AAV-CTL (CTL) and AAV-Y1 (OE) littermates were fed with HFD. **b** Gross view of CTL and OE mice. Scale bar, 0.5 cm. **c** Body weights of CTL and OE mice. **d** Weights and gross view of iWAT, BAT, and eWAT from AAV-CTL and AAV-Y1 mice. Scale bar, 0.5 cm. **e** H&E staining of adipose tissue. Scale bar, 50 μm. The white line indicated the average size. **f**–**h** O_2_ consumption **f**, CO_2_ generation **g**, and energy heat generation **h** of CTL and OE mice. White and gray areas in the graphs indicate day and night, respectively. **i** Rectal temperature in CTL and OE mice. **j** Serum concentrations of TG, CHO, LDL, and HDL in CTL and OE mice. **k**, **l** Glucose tolerance **k** and insulin tolerance (**l**) of CTL and OE mice. Data in **c**–**l** were presented as mean ± SEM (*n* = 4 biologically independent mice). ns, not significant, *P < 0. 05, **P < 0.01, ***P < 0.001, ****P < 0.0001, two-sided t-test. Source data are provided as a Source Data file.

To explore whether YTHDF1 overexpression affects obesity, direct bilateral injection of YTHDF1- or YFP-AAV into the inguinal fat pads of mice was performed, followed by HFD feeding for 8 weeks after 2 weeks of CD feeding (Fig. 7a). As expected, YTHDF1 expression alleviated HFD-induced obesity (Fig. 7b, c). A reduction in the weight of adipose tissue was observed in YTHDF1 expressing mice, resulting in smaller, multilocular adipocytes containing multiple lipid droplets (Fig. 7d, e). Metabolic cage experiments demonstrated that overexpression of YTHDF1 increased oxygen consumption, carbon dioxide generation, and energy heat generation during both light and dark cycles (Fig. 7f–h). The core temperature was much higher in YTHDF1-expressing mice (Fig. 7i). YTHDF1-expressing mice showed blunting of HFD-induced increases in TG, CHO, and LDL levels (Fig. 7j). Moreover, GTTs and ITTs demonstrated that glucose tolerance and insulin sensitivity were improved in YTHDF1-expressing mice (Fig. 7k, l). Collectively, these results demonstrated that WAT-specific YTHDF1 overexpression protected against HFD-induced obesity and metabolic disorders.

## Discussion

The m^6^A “writer” METTL3 promotes while the m^6^A “eraser” FTO inhibits thermogenesis of adipocytes^20–22^. In this study, we discovered that YTHDF1, an m^6^A “reader” protein, contributed to adipose tissue thermogenesis and systemic metabolism. The expression of YTHDF1, rather than other YTH family members, was reduced in obese mice and upregulated in beiging adipose tissue. *Ythdf1*^*cKO*^ mice gained more weight and showed aggravated insulin resistance and glucose intolerance after consumption of an HFD. By contrast, WAT-specific YTHDF1 overexpression tempered obesity-related symptoms. Moreover, YTHDF1 directly regulated the expression of BMP8B, an important stimulator of adipose tissue thermogenesis. Together, these findings demonstrated that YTHDF1 played important roles in promoting beiging and antagonizing HFD-induced obesity (Supplementary Fig. 7).

Both beige and brown adipose tissue can be activated to produce heat and take UCP1 as the main marker gene. However, the distinct feature of beige adipocytes, which distinguishes them from brown adipocytes, is their inducible and reversible thermogenic capacity in response to environmental stimuli. Emerging evidence suggests that an alternative thermogenic pathway may exist in beige fat and could contribute to the regulation of systemic energy expenditure and glucose homeostasis^7^. Intriguingly, studies showed that the metabolically active cells are mostly beige adipocytes in humans^29,30^. Gene expression analyses of multiple human fat depots suggest that the majority of UCP1^+^ fat cells in humans show beige fat characteristics instead of brown^30^, implying that beige adipocytes play an essential role in thermogenesis. Interestingly, YTHDF1 was shown to have different regulatory effects on UCP1 expression in various adipose tissues. Under cold stimulation, or HFD feeding, adipocyte-specific deletion of *Ythdf1* slightly affected UCP1 expression in BAT, but dramatically downregulated UCP1 in iWAT. This may be attributed to the fact that beige adipose tissue exhibits a more rapid response to stimulation, whereas BAT expresses constitutively high levels of UCP1. These findings proved that YTHDF1 is indispensable for beige adipose tissue, indicating its potential function in regulating systemic metabolism. Therefore, we used AAV to inject iWAT in situ for *Ythdf1* overexpression. Mice with *Ythdf1* overexpression in iWAT showed significant alleviation of obesity-related symptoms, including glucose intolerance, insulin resistance, and TG synthesis. These findings demonstrated that YTHDF1 specifically controlled the beiging of iWAT to regulate systemic metabolism.

As an m^6^A reader protein, YTHDF1 has been reported to enhance the translation of m^6^A-modified mRNAs^15^. Consistent with this, our data revealed a notable decrease in translational efficiency for m^6^A‐marked transcripts in *Ythdf1*-knockout mice compared with that in wild-type mice (Fig. 4d), suggesting that the effect of YTHDF1 on the translation of m^6^A-modified transcripts might be general for most m^6^A genes in adipocytes. Of note, not all m^6^A-modified mRNAs are YTHDF1 targets^15^, while some m^6^A-modified mRNAs are bound by YTHDF2. That is, YTHDF1 only regulates a group of m^6^A-modified mRNAs.

We observed decreased *Leptin* mRNA level in *Ythdf1*-KO iWAT (Supplementary Fig. 4E). Leptin is hypothesized to function as a negative feedback signal in the regulation of energy balance. It activates lipolysis and promote thermogenesis of adipose tissue^31,32^. Downregulation of *Leptin* mRNA might reflect the reduced thermogenesis in *Ythdf1*-KO iWAT. However, *Leptin* is not among the YTHDF1-regulated transcripts (Fig. 4g). That is, the translation of *Leptin* is not regulated by YTHDF1. Downregulation of *Leptin* might be an indirect effect of *Ythdf1*-KO. The regulation and function of *Leptin* in YTHDF1-promoted thermogenesis needs further investigation.

*Bmp8b* shows dramatical alteration after YTHDF1 knockout. Our data showed that YTHDF1 promotes *Bmp8b* translation mainly in iWAT, which could be explained by the finding that the percentage of m^6^A-modified *Bmp8b* mRNA is much lower in BAT than iWAT (Supplementary Fig. 5H). The detailed mechanism needs further investigation. BMP8B is an important ligand regulating adipose tissue thermogenesis and energy balance^33^. Autocrine signaling by BMP8B produced by beige/brown adipocytes enhance the energy dissipation of the cells^34^. Furthermore, adipocyte-specific *Bmp8b* overexpression enhances adipose tissue browning and thermogenesis^33,34^. Consistent with these reports, BMP8B expression was increased after cold stimulation. Importantly, BMP8B can rescue *Ythdf1*-deletion-induced impaired thermogenesis and beiging. Of note, we used the CDS region of *Bmp8b* for overexpression, which does not have m^6^A site and cannot be regulated by YTHDF1. Therefore, in some specific *Ythdf1*^*cKO*^ + BMP8B mice, the expression of BMP8B was much higher than *Ythdf1*^*CTL*^ mice, which induces higher UCP1 expression and thermogenesis. BMP8B acts as a pan-BMP/transforming growth factor β-receptor ligand and activates SMADs to regulate transcription^35,36^. BMP8B stimulation has also been demonstrated to activate SMAD1/5/8 in brown and beige adipocytes^33^. We also observed altered transcription in *Ythdf1*-KO tissue, which may be a secondary effect regulated by BMP8B/SMAD signaling. Thus, YTHDF1 may have applications in therapeutic strategies for the management of obesity-associated metabolic diseases.

## Methods

### Ethics statement

All animal studies were performed in compliance with the Guide for the Care and Use of Laboratory Animals by the Medical Experimental Animal Care Commission of Zhejiang University. All animal studies used the protocol that has been approved by the Medical Experimental Animal Care Commission of Zhejiang University (ZJU20220512).

### Mouse experiments

Mice were maintained and bred in specific pathogen-free conditions at the Animal Center of Zhejiang University. All animal studies were performed in compliance with the Guide for the Care and Use of Laboratory Animals by the Medical Experimental Animal Care Commission of Zhejiang University. Only adult male mice were used in our experiments. For each experiment, about 3–6 mice were used for each group. Randomization and blinding were used for animal studies. All mice were housed in a pathogen-free and climate-controlled environment (22–25 °C, 40–60% humidity) with a 12-h light–dark cycle that provided free access to food and water unless stated otherwise.

Conditional *Ythdf1* KO allele (*Ythdf1*^*CTL*^) was generated by Cyagen Biosciences (China). The fourth exon of *Ythdf1* was targeted with flanking LoxP sites. AdipoQ-Cre mice were obtained from the Jackson Laboratory. All of the primers for PCR genotyping were listed in Supplementary Table1.

For obese mice model, C57BL/6 J male mice at 8–10-week-old were fed with HFD (60% fat as kcal, D12492, ResearchDiet Inc, New Brunswick, NJ) for 8–12 weeks as indicated in Figures. Chow diet (10% fat as kcal, D12450J, ResearchDiet Inc) was used as control. For cold exposure, mice were individually housed in plastic cages at 4 °C for 7 days. For thermoneutral condition, mice were individually housed in plastic cages at 29 °C. Mice were intraperitoneally injected with CL316,243(Sigma) at 1 mg/kg/day for seven consecutive days. To exclude the BAT function, interscapular BAT was surgically removed^37,38^. The OCR of adipose tissues was performed using Clark-type oxygen electrodes (Strathkelvin Instruments)^39^. For histology analysis, adipose tissues were rinsed with DPBS and fixed in 10% formalin. Hematoxylin and eosin (H&E) staining and immunohistochemistry were performed.

### Cells and reagents

3T3-L1 pre-adipocytes (CL-173) and HEK293T cells (CRL-3216) are obtained from ATCC and cultured in DMEM medium (HyClone) supplemented with 10% fetal bovine serum (Thermo Fisher Scientific). The primary preadipocytes were digested in Dispase II (Sigma-Aldrich) and cultured in DMEM/F12 medium (Gibco)^40^. The seahorse assay was performed using Seahorse XF96 Analyzer (Agilent).

### Plasmids construction

To construct shRNA for YTHDF1-shRNA virus, shRNA oligos of YTHDF1 were cloned to the lentiviral vector pLKO.1. Transgenes encoding YFP or YTHDF1 were inserted into the multiple cloning sites of rAAV2 vector. To achieve adipose tissue-specific expression, the promoter in the vector was replaced with AdipoQ promoter. The oligos for shRNA construction are listed in Supplementary Table 1.

### RNA isolation and quantitative RT-PCR

Total RNA was isolated from various adipose tissues of mice or cells using TRIzol reagent (Life Technologies). Reverse transcription of RNA sample with M-MLV reagent (Takara) using random primers. Real-time PCR was performed using the SYBR Green I master mix (Takara) on a Light Cycler 480 real-time PCR system (Roche). Relative gene expression was normalized for the ACTB or GAPDH reference gene and was calculated using the 2(−ΔΔCT) method. RT-qPCR primers are listed in Supplementary Table 2.

### Immunoblotting

Cells or adipose tissues were lysed in RIPA lysis buffer (Beyotime, China). Total protein under denaturing conditions was separated by sodium dodecyl sulfate-polyacrylamide gel (SDS-PAGE) and transferred to PVDF membranes (Millipore). Membranes were blocked and incubated with primary antibodies, followed by incubation with the secondary antibody and chemiluminescent detection system (Bio-Rad). Anti-YTHDF1 (Proteintech, 17479-1-AP), Anti-YTHDF2 (Proteintech, 24744-1-AP), Anti-YTHDF3 (Proteintech, 25537-1-AP), Anti-UCP1 (Proteintech, 23673-−1-AP), Anti-MCP1 (Abcam, ab214819), Anti-BMP8B (Shanghai Huzhen, HZ-12837R) were used for immunoblotting at 1:1000 dilution. Anti-Alpha-Tubulin (Abclonal, A6830) and anti-ACTB (Proteintech, 66009-1-Ig) were used for immunoblotting at 1:3000 dilution.

### Polysome Profiling

Cells or tissues were lysed in polysome lysis buffer (10 mM HEPES, pH 7.4, 100 mM KCl, 5 mM MgCl_2_, 100 μg/ml CHX, 5 mM DTT, and 1% Triton X‐100) and centrifuged. The supernatant was centrifuged in gradient sucrose (Beckman, rotor SW41Ti), fractioned (BioCamp), and collected (FC203B, Gilson). Total RNA from the indicated fractions were isolated by TRIzol reagent for RT-qPCR analysis^41^.

### Virus packaging

The lentivirus was packaged in HEK293T cells with helper vector pMD2G and psPAX2 by transfecting cells with indicated constructs. The AAV was packaged with pAAV-RC2 and pHelper in AAV293 cells.

### rAAV injection to inguinal WAT

A total of 6-week-old mice were anesthetized with 2% isoflurane in O_2_. After anesthesia was fully induced, mice were injected with rAAV (1.0 × 10^12^ vg per 20 μl phosphate buffered saline) with a 0.3 cc, 30 G insulin syringe^42^. Finally, the wound was closed with 4-0 PDS II FS-2 suture.

### Metabolic analysis

Body weight and food intake were measured weekly. For GTT, mice were intraperitoneally injected of D-glucose (2 g/kg body weight, 1 g/kg body weight for obese mice) after overnight starvation. For ITT, mice were intraperitoneally injected with insulin (0.7 U/kg body weight, 1.5 U/kg body weight for obese mice) after 5 hr fasting. Serum glucose levels were determined in tail blood samples at 0, 15, 30, 60, 90, and 120 min after glucose or insulin injection using a glucometer (Accu-Chek, Roche). Core body temperature was measured intra-rectally at around 4 p.m. The energy expenditure, including O_2_ consumption, CO_2_ generation, and energy heat generation, were monitored using Promethion High-Definition Behavioral Phenotyping System for Mice (Sable Systems International).

### Biochemical analysis of plasma

Blood samples were obtained from cardiac puncture, and plasma was collected after centrifugation for 15 min at 3000 rpm at 4 °C. Total cholesterol, triglyceride, low-density lipoprotein cholesterol and high-density lipoprotein cholesterol were measured by using the assay kits were from HaoKe Biotech Co., Ltd. (Shanghai, China).

### RNA-seq

The polyadenylated RNA was enriched from total RNA using the Dynabeads Oligo(dT)25 (Invitrogen, USA) and fragmented into ~100-nucleotide-long fragments using RNA fragmentation reagent (Ambion, AM8740). Fragmented RNA samples were used for library construction and high‐throughput sequencing.

### m^6^A-seq

Fragmented mRNA was incubated with m^6^A antibody-coated beads for 6 h at 4 °C. The immunoprecipitation complex was digested with Proteinase K at 55 °C for 1 h. RNA was then extracted using TRIzol reagent and used for library construction.

### Ribo-seq

iWAT tissue was treated with polysome lysis buffer. After centrifugation, the supernatant was digested with *E. coli* RNase I (Ambion) for 1 h. Ribosome-protected fragments were collected by ultracentrifugation. RNA was extracted using TRIzol reagent. The library was constructed with small RNA library construction kit (NEB)^41^.

### Statistical analysis

Illumina Casava1.7 software used for basecalling. Sequenced reads were trimmed for adaptor sequence, and masked for low-complexity or low-quality sequence, then mapped to mm8 whole genome using Hisat2 v2.2.1^41^. Statistical analysis was performed using GraphPad Prism 8 software (GraphPad Software, Inc.). Data were presented as mean ± standard errors of the means (SEM). P values were calculated using a two‐tailed t‐test unless stated otherwise. Asterisks denote statistical significance (*P < 0.05; **P < 0.01; ***P < 0.001; ****P < 0.0001).

### Statistics and reproducibility

All of the experiments had repeated at least 3 times independently with similar results.

### Reporting summary

Further information on research design is available in the Nature Portfolio Reporting Summary linked to this article.

## Supplementary information

Supplementary information

Reporting summary

## Data Availability

The sequencing data of m^6^A-seq data used in this study are available in Gene Expression Omnibus under accession code GSE178884. The sequencing data of RNA-seq and Ribo-seq data used in this study are available in Gene Expression Omnibus under accession code GSE197173. Source data are provided with this paper. The human sequencing data of RNA-seq data reused in this study are available in Gene expression omnibus under accession code GSE156906^43^, GSE162653^44^, and GSE110729^45^. All other data generated or analysed during this study are included in this published article (and its supplementary information files). Source data are provided with this paper.

## Source data

Source data
