# Supplementary material for: Adipocyte YTH N(6)-methyladenosine RNA-binding protein 1 protects against obesity by promoting white adipose tissue beiging in male mice
Source: Nat Commun. 2023 Mar 13;14:1379. doi: 10.1038/s41467-023-37100-z (PMC10011519; doi:10.1038/s41467-023-37100-z)
Supplement: Supplementary file 1 — Supplementary information [file 41467_2023_37100_MOESM1_ESM.pdf]

Adipocyte YTH N(6)-methyladenosine RNA-binding protein 1 protects against obesity by promoting white adipose tissue beiging in male mice

Sujun Yan[1], Xiaoling Zhou[1], Canlan Wu[1], Yunyi Gao[1], Yu Qian[1], Jingyu Hou[1], Renxiang Xie[1], Bing Han[1], Zhanghui Chen[5,*], Saisai Wei[1,2,*], Xiangwei Gao[1,3,4,*].

1, Sir Run-Run Shaw Hospital, Institute of Environmental Medicine, Zhejiang University School of Medicine, Hangzhou 310058, China.

2, Key Laboratory of Laparoscopic Technology of Zhejiang Province, Department of General Surgery, Sir Run-Run Shaw Hospital, Zhejiang University School of Medicine, Hangzhou 310016, China.

3, Department of Clinical Laboratory, Sir Run Run Shaw Hospital, Zhejiang University School of Medicine, Hangzhou 310016, China.

4, Bioelectromagnetics Laboratory, Zhejiang University School of Medicine, Hangzhou 310058, China.

5, Zhanjiang Institute of Clinical Medicine, Zhanjiang Central Hospital, Guangdong Medical University, Zhanjiang, 524037, China.

* Correspondence: Xiangwei Gao, xiangweigao@zju.edu.cn; or Zhanghui Chen, zhc34@gdmu.edu.cn; or Saisai Wei, saisaiwei@zju.edu.cn.

SUPPLEMENTARY INFORMATION

Supplementary Figures 1-7

Supplementary Tables 1-2

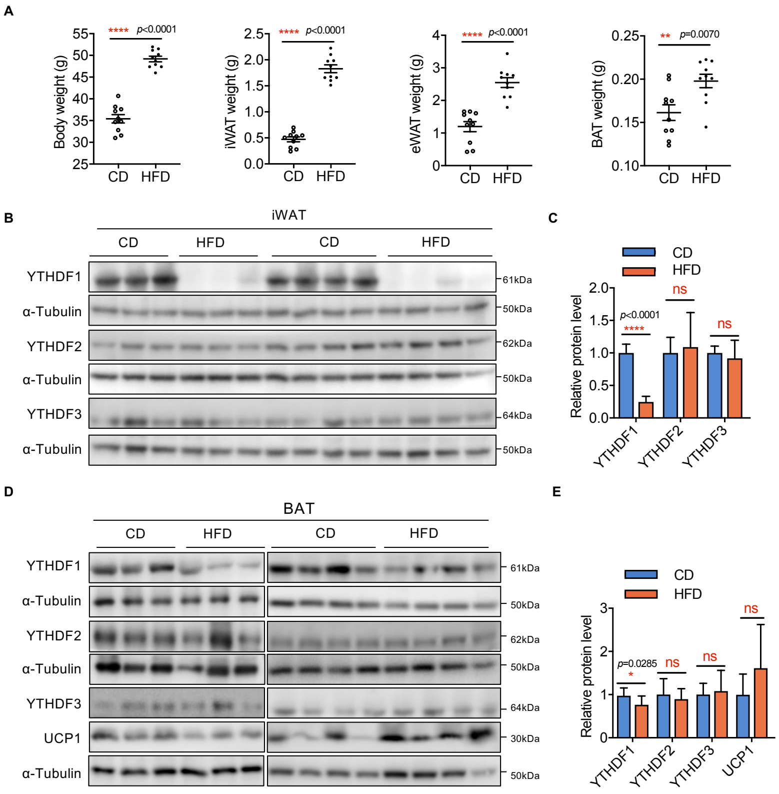

Supplementary Fig. 1 Adipose YTHDF1 expression is reduced in obesity. **A** Weights of Body, iWAT, eWAT, and BAT of CD- and HFD-fed mice (n = 10). Individual values and mean ± SEM were shown. **B, D** Immunoblot analysis of YTH family proteins in iWAT (**B**) and BAT (**D**) of CD- and HFD-fed mice. **C, E** The quantification of protein bands. Data in **A,C,E** were presented as mean ± SEM (n = 10). ns, not significant * $P < 0.05$, ** $P < 0.01$, **** $P < 0.0001$, two-sided t-test. Source data are provided as a Source Data file.

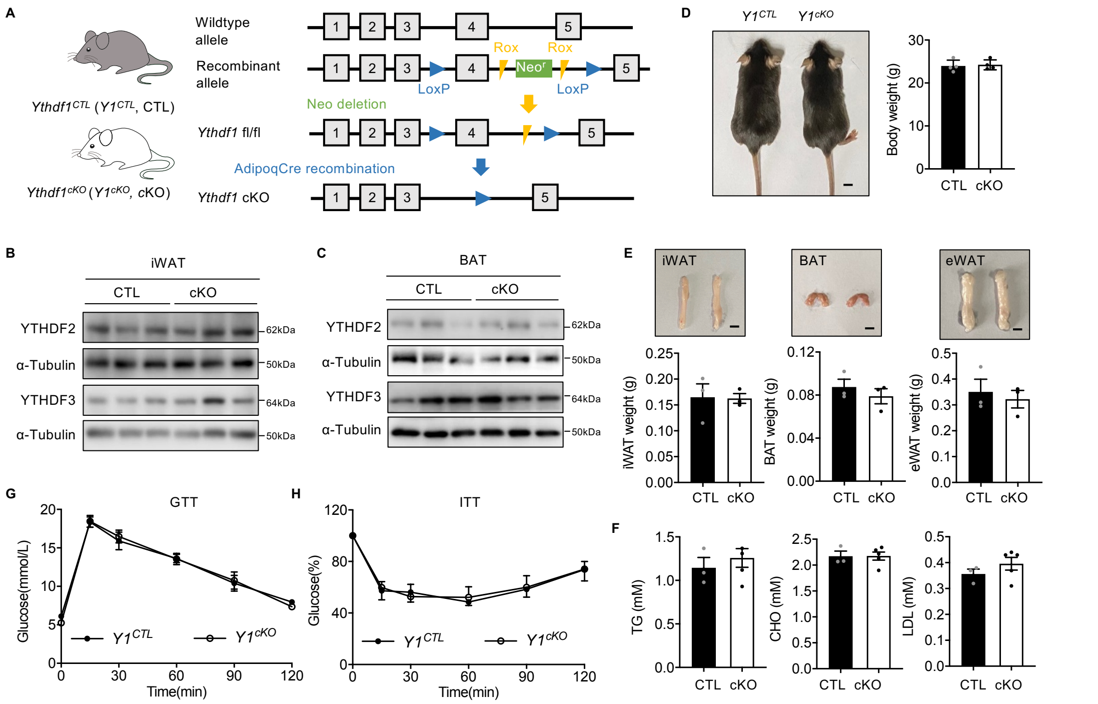

Supplementary Fig. 2 Adipocyte-specific knockout of *Ythdf1* does not affect weight gain under normal condition. **A** Schematic of the *Ythdf1* deletion mouse. **B, C** Expression of YTHDF2/3 in iWAT (**B**) and BAT (**C**) of $Y1^{CTL}$ and $Y1^{cKO}$ mice. **D** Gross view of mice ($Y1^{CTL}$ and $Y1^{cKO}$ mice) under normal condition. Body weights of $Y1^{CTL}$ and $Y1^{cKO}$ littermates at 8 weeks of age (n = 4). **E** Weights and gross view of iWAT, BAT, and eWAT from $Y1^{CTL}$ and $Y1^{cKO}$ mice at 8 weeks of age (n = 3). **F** Serum concentrations of TG, CHO, and LDL in $Y1^{CTL}$ and $Y1^{cKO}$ mice (n = 3). **G, H** Glucose tolerance (**G**) and insulin tolerance (**H**) at 8 weeks of age (n = 4). Data in **D-H** were presented as mean ± SEM. Source data are provided as a Source Data file.

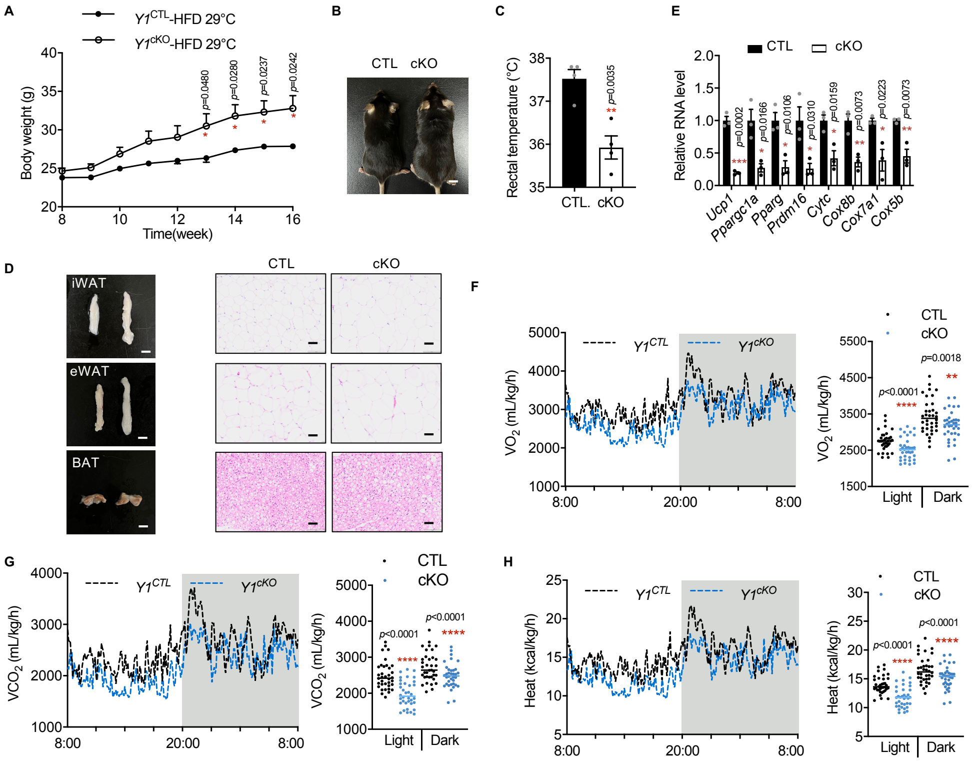

Supplementary Fig. 3 Deletion of *Ythdf1* exacerbates obesity and reduces thermogenesis under thermoneutral condition. **A-H** $Y1^{CTL}$ and $Y1^{cKO}$ littermates were fed under thermoneutral condition (29°C). **A** Body weights of $Y1^{CTL}$ and $Y1^{cKO}$ mice. **B** Gross view of $Y1^{CTL}$ and $Y1^{cKO}$ mice. Scale bar, 0.5 cm. **C** Rectal temperature in $Y1^{CTL}$ and $Y1^{cKO}$ mice. **D** Gross view and H&E staining of iWAT, eWAT, and BAT from $Y1^{CTL}$ and $Y1^{cKO}$ mice. Scale bar, 0.5 cm and 50 μm. **E** Thermogenesis genes level in $Y1^{CTL}$ and $Y1^{cKO}$ mice. **F, G, H** O_2 consumption (**F**), CO_2 generation (**G**), and energy heat generation (**H**) of $Y1^{CTL}$ and $Y1^{cKO}$ mice fed with HFD. White and gray areas in the graphs indicate day and night, respectively. Data in **A,C,E-H** were presented as mean ± SEM (n = 4 biologically independent mice). * $P < 0.05$, ** $P < 0.01$, *** $P < 0.001$, **** $P < 0.0001$, two-sided t-test. Source data are provided as a Source Data file.

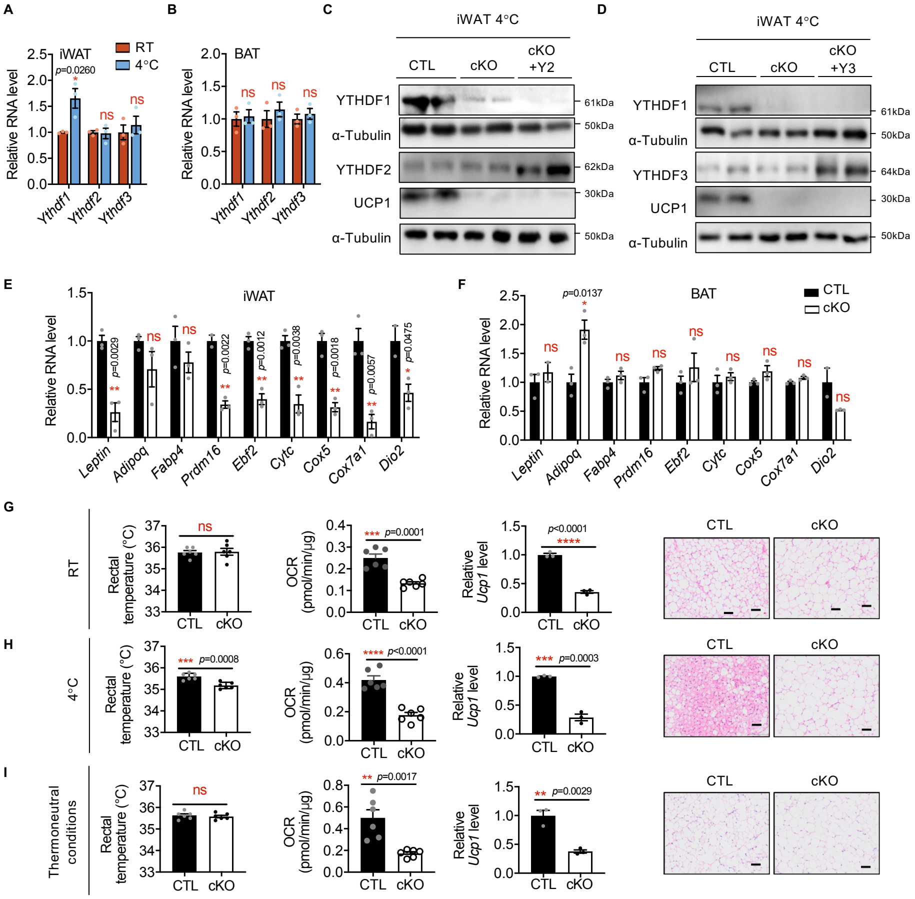

Supplementary Fig. 4 Deletion of *Ythdf1* impairs WAT beiging. **A, B** mRNA levels of YTH family proteins expression in iWAT (**A**) and BAT (**B**) from mice housed at room temperature (RT) or 4°C (n=3 biologically independent mice). **C, D** UCP1 expression in iWAT of $Y1^{CTL}$ and $Y1^{cKO}$ mice with or without YTHDF2/3 overexpression after cold exposure. **E, F** mRNA levels of adipogenic genes (*Lept, adipoq, Fabp4*), BAT-specific genes (*Prdm16, Ebf2*), and mitochondrial specific genes (*Cytc, Cox5, Cox7, Dio2*) in iWAT (**E**) and BAT (**F**) from $Y1^{\mathrm{CTL}}$ and $Y1^{\mathrm{cKO}}$ mice (n=3 biologically independent mice). **G-I** Rectal temperature, the oxygen consumption rates (OCR) of iWAT, and the *Ucp1* expression were measured under RT (**G**), cold (**H**), and thermoneutral conditions (29°C) (**I**) in $Y1^{CTL}$ and $Y1^{cKO}$ mice (n = 6). H&E staining of iWAT was shown in right panel. Scale bar, 50 µm. All the data were presented as mean ± SEM. * $P < 0.05$, ** $P < 0.01$, *** $P < 0.001$, **** $P < 0.0001$, two-sided t-test. Source data are provided as a Source Data file.

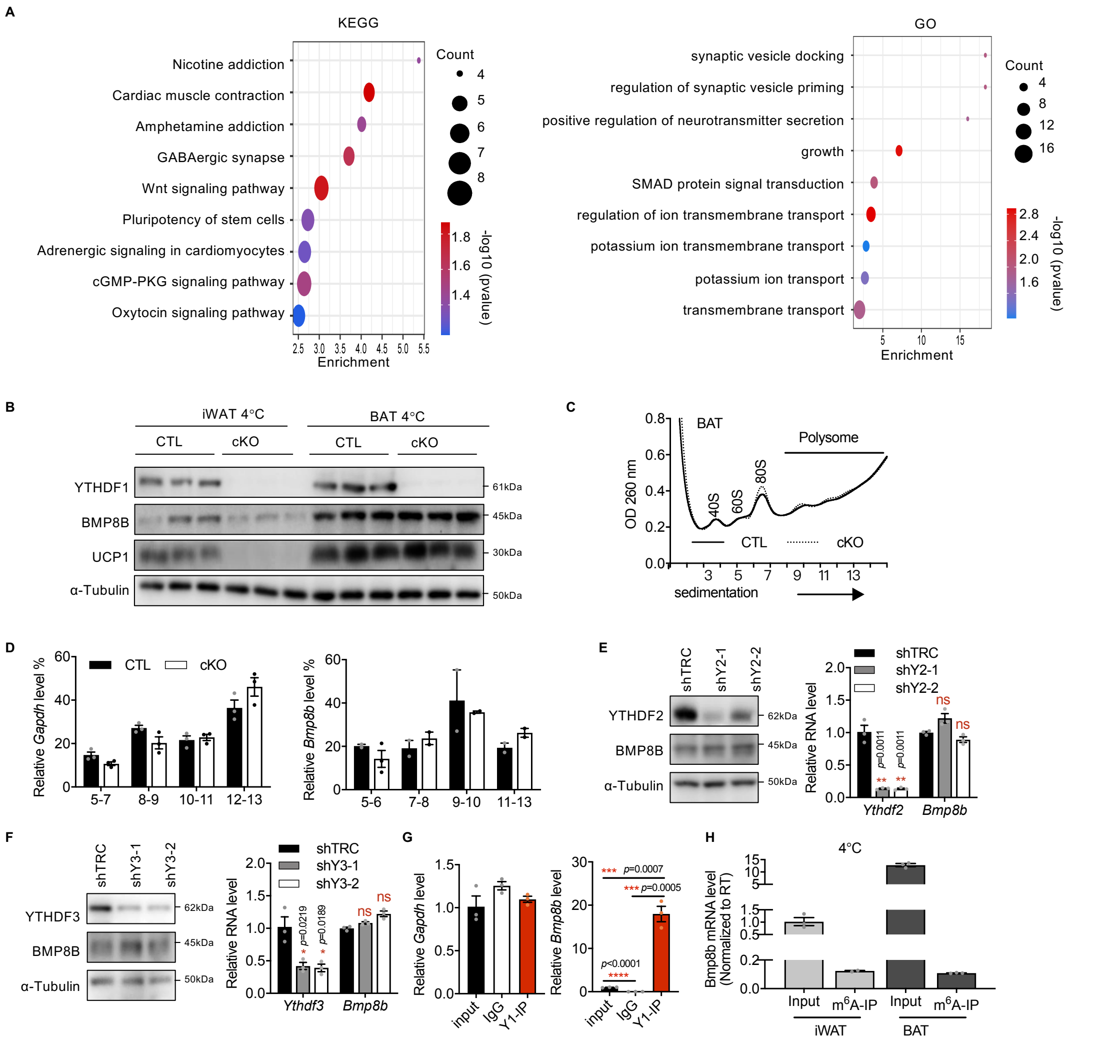

Supplementary Fig. 5 Identification of YTHDF1-regulated mRNAs. **A** Top KEGG and GO analysis terms enriched for translational upregulated transcripts. **B** The protein level of BMP8B in iWAT and BAT from $Y1^{CTL}$ and $Y1^{cKO}$ mice after cold exposure. **C** Polysome profiles of BAT from $Y1^{CTL}$ and $Y1^{cKO}$ mice. **D** The distributions of *Gapdh* and *Bmp8b* in polysome fractions. Data were presented as mean ± SEM (n = 3). **E, F** The expression of YTHDF2/3 and BMP8B in 3T3-L1 cells with or without *Ythdf2/3* knockdown (n=3 biologically independent samples). **G** RIP-qPCR analysis of the association of *Bmp8b* with YTHDF1 in iWAT of cold-treated mice. **H** Methylated RNA immunoprecipitation (MeRIP)-qPCR analysis of m^6A levels of *Bmp8b* mRNA in iWAT and BAT from cold-treated mice. **G, H** n=3 biologically independent mice. Data in **E-H** were presented as mean ± SEM. * $P < 0.05$, ** $P < 0.01$. All of the P-values are determined by unpaired two-sided t-test. Source data are provided as a Source Data file.

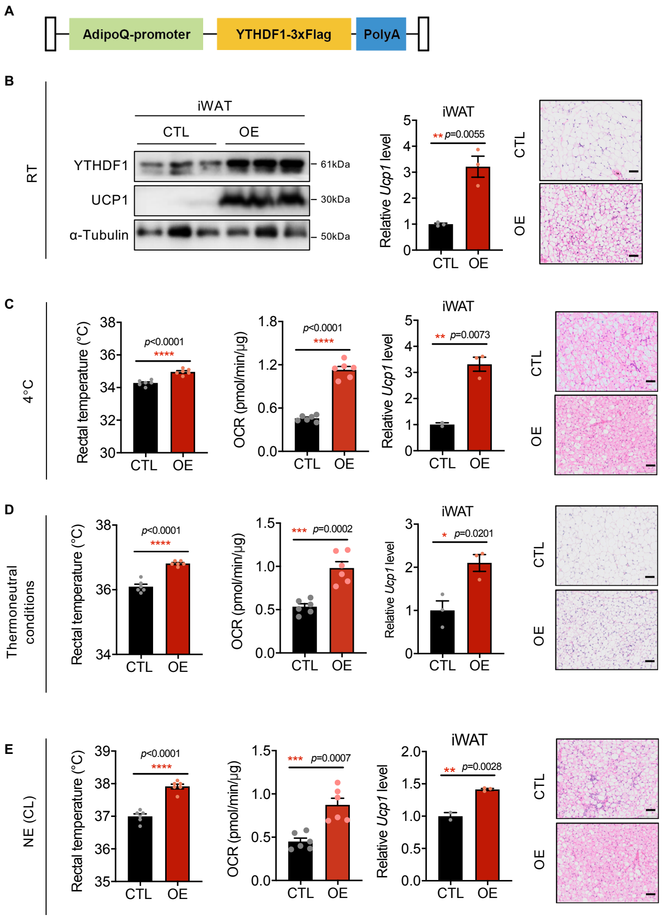

Supplementary Fig. 6 Overexpression YTHDF1 enhances thermogenic capacity. **A** Schematic of AdipoQ-promoter driven YTHDF1 expression. **B-E** Mice expressing YFP (CTL) or YTHDF1 (OE) were treated under RT (**B**), 4°C (**C**), thermoneutral condition (29°C) (**D**), or CL stimulation (**E**). The UCP1 expression, rectal temperature, OCR, and H&E staining of iWAT were determined. Scale bar, 50 μm. All the data were presented as mean ± SEM (n = 6 biologically independent mice). * $P < 0.05$, ** $P < 0.01$, *** $P < 0.001$, **** $P < 0.0001$, two-sided t-test. Source data are provided as a Source Data file.

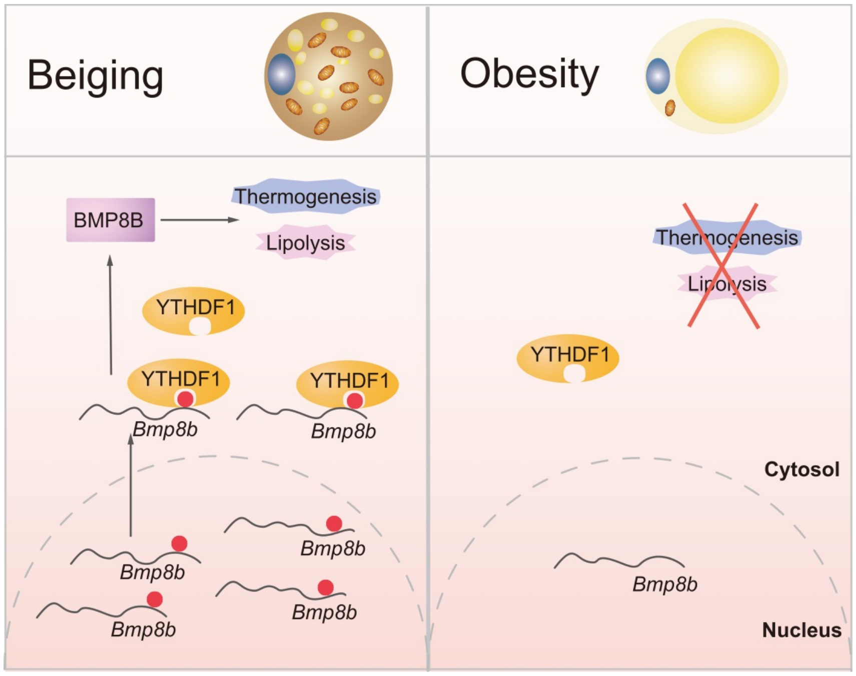

Supplementary Fig. 7 A proposed model of YTHDF1-promoted WAT beiging.

Supplementary Table 1. Sequences of primers used in this study

Primers for plamids construction	
Bmp8b-CDS-F	5'-AGATCGCCGTGTAATTCTAGAGTGGTCCAAGAGCAC-3'
Bmp8b-CDS-R	5'-GCCGGCCGCCCCGACTCTAGAACAGCACACCTTGGG-3'
Bmp8b-3'UTR-1-F	5'-AGATCGCCGTGTAATTCTAGAGTCCCTGCCCAACAG-3'
Bmp8b-3'UTR-1-R	5'-<br>GCCGGCCGCCCCGACTCTAGATTTTTTAAACTCTTCTCTAG<br>AAATCCCA-3'
Bmp8b-3'UTR-2-F	5'-AGATCGCCGTGTAATTCTAGACCCAGAACAGCAGCC-3'
Bmp8b-3'UTR-2-R	5'-GCCGGCCGCCCCGACTCTAGACTACTGGAGGGTCCC-3'
YTHDF1-F	5'-<br>TGGCAAAGAATTGGATCCGCCACCATGTCGGCCACCAGCG<br>TG-3'
YTHDF1-R	5'-TTGTTTGTTTCGACTCTGCCGT-3'
Primers for shRNA plamids construction	
shTRC	5'-CAACAAGATGAAGAGCACCAA-3'
sh*Ythdf1*	5'-GCTGAAGATTATCGCTTCCTA-3'
Pimers for genotyping	
Ythdf1-F	GCCAGTAGTGTGGTGTTTTGAG
Ythdf1-R	AGAGCTTCCAACTGCTATGTGG
Adipoq-Cre-F	ACGGACAGAAGCATTTTCCA
Adipoq-Cre-R	ACGGACAGAAGCATTTTCCA

Supplementary Table 2. Sequences of primers used in this study

Pimers for RT-qPCR	
Ythdf1-F	5’-ACAGTTACCCCTCGATGAGTG-3’
Ythdf1-R	5’-GGTAGTGAGATACGGGATGGGA-3’
Ythdf2-F	5’-GAGCAGAGACCAAAAGGTCAAG-3’
Ythdf2-R	5’-CTGTGGGCTCAAGTAAGGTTC-3’
Ythdf3-F	5’-CATAGGGCAACAGAGGAAACAG-3’
Ythdf3-R	5’-ATCTCCAGCCGTGGACCAT-3’
Bmp8b-F	5’-TCCACCAACCACGCCACTAT-3’
Bmp8b-R	5’-CAGTAGGCACACAGCACACCT-3’
Ucp1-F	5’-GAGGTCGTGAAGGTCAGAAT-3’
Ucp1-R	5’-CTGTGGTGGCTATAACTCTGTAA-3’
Dio2-F	5’-GGTGGTCAACTTTGGTTCAGCC-3’
Dio2-R	5’-AAGTCAGCCACCGAGGAGAACT-3’
HSL-F	5’-GCTGGGCTGTCAAGCACTGT-3’
HSL-R	5’-GTAACTGGGTAGGCTGCCAT-3’
Cidea-F	5’-GGTGGACACAGAGGAGTTCTTTC-3’
Cidea-R	5’-CGAAGGTGACTCTGGCTATTCC-3’
Ppargc1a-F	5’-CCCTGCCATTGTTAAGACC-3’
Ppargc1a-R	5’-TGCTGCTGTTCCTGTTTTC-3’
Pparg-F	5’-GGAAGACCACTCGCATTCCTT-3’
Pparg-R	5’-GTAATCAGCAACCATTGGGTCA-3’
Prdm16-F	5’-CCACCAGCGAGGACTTCAC-3’
Prdm16-R	5’-GGAGGACTCTCGTAGCTCGAA-3’
Adrb3-F	5’-TCTCTGGCTTTGTGGTCGGA-3’
Adrb3-R	5’-GTTGGTTATGGTCTGTAGTCTCG-3’
Cox8b-F	5’-GAACCATGAAGCCAACGACT-3’
Cox8b -R	5’-GCGAAGTTCACAGTGGTTCC-3’
Leptin-F	5’-GGGCTTCACCCCATTCTGA-3’
Leptin-R	5’-TGGCTATCTGCAGCACATTTTG-3’

Adipoq-F	5’-GTTCCCAATGTACCCATTCGC-3’
Adipoq-R	5’-TGTTGCAGTAGAACTTGCCAG-3’
Fabp4-F	5’-GACGACAGGAAGGTGAAGAG-3’
Fabp4-R	5’-ACATTCCACCACCAGCTTGT-3’
Ebf2-F	5’-GCT GCG GGA ACC GGA ACG AGA-3’
Ebf2-R	5’-ACA CGA CCT GGA ACC GCC TCA-3’
Cytc-F	5’-CCAAATCTCCACGGTCTGTTC-3’
Cytc-R	5’-ATCAGGGTATCCTCTCCCCAG-3’
Cox5b-F	5’-GCTGCATCTGTGAAGAGGACAAC-3’
Cox5b-R	5’-CAGCTTGTAATGGGTTCCACAGT-3’
Cox7a1-F	5’-CAG CGT CAT GGT CAG TCT GT-3’
Cox7a1-R	5’-AGA AAA CCG TGT GGC AGA GA-3’
ATGL-F	5’-GAGGAATGGCCTACTGAACCAA-3’
ATGL-R	5’-AGGCTGCAATTGATCCTCCTC-3’
Pref1-F	5’-GACCCACCCTGTGACCCC-3’
Pref1-R	5’-CAGGCAGCTCGTGCACCCC-3’
Adipsin-F	5’-CGTACCATGACGGGGTAGTC-3’
Adipsin-R	5’-ATCCGGTAGGATGACACTCG-3’
Luciferase-F	5’-CTAAGGAAGTCGGGGAAGCG-3’
Luciferase-R	5’-ATCCCCCTCGGGTGTAATCA-3’
