# Supplementary material for: Adipocyte YTH N(6)-methyladenosine RNA-binding protein 1 protects against obesity by promoting white adipose tissue beiging in male mice
Source: Nat Commun. 2023 Mar 13;14:1379. doi: 10.1038/s41467-023-37100-z (PMC10011519; doi:10.1038/s41467-023-37100-z)
Supplement: Supplementary file 2 — Reporting summary [file 41467_2023_37100_MOESM2_ESM.pdf]

nature portfolio

Corresponding author(s): Xiangwei Gao, Ph.D.

Last updated by author(s): Feb 2, 2023

Reporting Summary

Nature Portfolio wishes to improve the reproducibility of the work that we publish. This form provides structure for consistency and transparency in reporting. For further information on Nature Portfolio policies, see our Editorial Policies and the Editorial Policy Checklist.

Statistics

For all statistical analyses, confirm that the following items are present in the figure legend, table legend, main text, or Methods section.

n/a	Confirmed	
	x	The exact sample size (*n*) for each experimental group/condition, given as a discrete number and unit of measurement
	x	A statement on whether measurements were taken from distinct samples or whether the same sample was measured repeatedly
	x	The statistical test(s) used AND whether they are one- or two-sided<br>*Only common tests should be described solely by name; describe more complex techniques in the Methods section.*
x		A description of all covariates tested
x		A description of any assumptions or corrections, such as tests of normality and adjustment for multiple comparisons
	x	A full description of the statistical parameters including central tendency (e.g. means) or other basic estimates (e.g. regression coefficient) AND variation (e.g. standard deviation) or associated estimates of uncertainty (e.g. confidence intervals)
	x	For null hypothesis testing, the test statistic (e.g. *F*, *t*, *r*) with confidence intervals, effect sizes, degrees of freedom and *P* value noted<br>*Give P values as exact values whenever suitable.*
x		For Bayesian analysis, information on the choice of priors and Markov chain Monte Carlo settings
x		For hierarchical and complex designs, identification of the appropriate level for tests and full reporting of outcomes
x		Estimates of effect sizes (e.g. Cohen's *d*, Pearson's *r*), indicating how they were calculated

Our web collection on statistics for biologists contains articles on many of the points above.

Software and code

Policy information about availability of computer code

Data collection	Illumina Casava1.7 software used for basecalling.
Data analysis	Sequenced reads were trimmed for adaptor sequence, and masked for low-complexity or low-quality sequence, then mapped to mm8 whole genome using Hisat2 v2.2.1 . Statistical analysis was performed using GraphPad Prism 8 software (GraphPad Software, Inc.).

For manuscripts utilizing custom algorithms or software that are central to the research but not yet described in published literature, software must be made available to editors and reviewers. We strongly encourage code deposition in a community repository (e.g. GitHub). See the Nature Portfolio guidelines for submitting code & software for further information.

Data

Policy information about availability of data

All manuscripts must include a data availability statement. This statement should provide the following information, where applicable:

- Accession codes, unique identifiers, or web links for publicly available datasets
- A description of any restrictions on data availability
- For clinical datasets or third party data, please ensure that the statement adheres to our policy

The sequencing data of m6A-seq data used in this study are available in Gene Expression Omnibus under accession code GSE178884 (https://www.ncbi.nlm.nih.gov/geo/query/acc.cgi?acc=GSE178884). The sequencing data of RNA-seq and Ribo-seq data used in this study are available in Gene Expression Omnibus under accession code GSE197173 (https://www.ncbi.nlm.nih.gov/geo/query/acc.cgi?acc=GSE197173). The human sequencing data of RNA-seq data used in this study are available in Gene expression omnibus under accession code GSE156906 43 (https://www.ncbi.nlm.nih.gov/geo/query/acc.cgi?acc=GSE156906), GSE162653 44

(https://www.ncbi.nlm.nih.gov/geo/query/acc.cgi?acc=GSE162653), and GSE110729 45 (https://www.ncbi.nlm.nih.gov/geo/query/acc.cgi?acc=GSE110729). Source data are provided with this paper.

Human research participants

Policy information about studies involving human research participants and Sex and Gender in Research.

Reporting on sex and gender	N/A
Population characteristics	N/A
Recruitment	N/A
Ethics oversight	N/A

Note that full information on the approval of the study protocol must also be provided in the manuscript.

Field-specific reporting

Please select the one below that is the best fit for your research. If you are not sure, read the appropriate sections before making your selection.

☒ Life sciences ☐ Behavioural & social sciences ☐ Ecological, evolutionary & environmental sciences

For a reference copy of the document with all sections, see nature.com/documents/nr-reporting-summary-flat.pdf

Life sciences study design

All studies must disclose on these points even when the disclosure is negative.

Sample size	For each experiment, about 3–6 mice were used for each group. In order to minimize the influence of individual differences in mice, we use littermates. The sample size folllowing common standards there or more biological replicates. All sample size are listed in the figure legend. P values were calculated using a two-tailed t-test unless stated otherwise.
Data exclusions	No data were excluded from the analyses.
Replication	Three biological replicates were used for verifying the reproducibility of the experimental findings. All the attempts at replication were successful. We have added the statement in the Figure legends and the source data in the source data file.
Randomization	In order to minimize the influence of individual differences in mice, we use littermates for experiments. Littermates were randomly allocated into experimental groups.
Blinding	Investigators were blinded to group allocation during data collection and analysis.

Reporting for specific materials, systems and methods

We require information from authors about some types of materials, experimental systems and methods used in many studies. Here, indicate whether each material, system or method listed is relevant to your study. If you are not sure if a list item applies to your research, read the appropriate section before selecting a response.

Materials & experimental systems

n/a	Involved in the study	
☐	☒	Antibodies
☐	☒	Eukaryotic cell lines
☒	☐	Palaeontology and archaeology
☐	☒	Animals and other organisms
☒	☐	Clinical data
☒	☐	Dual use research of concern

Methods

n/a	Involved in the study	
☒	☐	ChIP-seq
☒	☐	Flow cytometry
☒	☐	MRI-based neuroimaging

Antibodies

Antibodies used	Supplier name/Catalog number/Clone name/Lot number/dillution<br>Proteintech/17479-1-AP/anti-YTHDF1/00092060/11:1000

	Proteintech/24744-1-AP/anti-YTHDF2/00102169/1:1000<br>Proteintech/25537-1-AP/anti-YTHDF3/00049687/1:1000<br>Abclonal A6830/anti-Alpha-Tubulin/9100012001/1:1000<br>Proteintech/66009-1-Ig/anti-ACTB/1004156/1:1000<br>Shanghai Huzhen/HZ-12837R/anti-BMP8B/2201124/1:1000<br>Proteintech/23673--1-AP/anti-UCP1/00084987/1:1000<br>Abcam/ab214819/anti-MCP1/GR3334997-2/1:1000<br>Synaptic Systems/202 003/anti-m6A/2-111/1:1000
Validation	Clone name/Species/Applications<br>anti-YTHDF1/human, mouse, rat/CoIP, IF, IHC, IP, RIP, WB<br>anti-YTHDF2/human, mouse, rat/ChIP, CLIP, CoIP, IF, IP, RIP, WB<br>anti-YTHDF3/chicken, human, mouse/IF, IHC, RIP, WB<br>anti-Alpha-Tubulin/human, mouse, rat/IP, IF, IHC, WB<br>anti-ACTB/human, mouse, rat, hamster, zebrafish, monkey, dog/ChIP, CoIP, IF, IHC, IP,WB<br>anti-BMP8B/human, mouse, rat/IF, IHC, ICC, WB, ELISA<br>anti-UCP1/human, mouse, rat, chicken, dog, pig, cow, sheep/IF, IHC, WB<br>anti-MCP1/human/ICC,WB,IP<br>anti-m6A/human, mouse, rat, eukaryotes, prokaryotes/ IP, ICC, IHC, IHC-P, WB, ELISA

Eukaryotic cell lines

Policy information about cell lines and Sex and Gender in Research

Cell line source(s)	HEK293T cell line (Human Embryonic Kidney Cells, ATCC,CRL-3216); 3T3-L1(Mouse embryo-derived NIH 3T3 cell line, ATCC,CL-173)
Authentication	Cell lines were authenticated by DNA profiling assays (STR) .
Mycoplasma contamination	Cell lines were confirmed negative for mycoplasma contamination
Commonly misidentified lines<br>(See ICLAC register)	The study did not use commonly misidentified cell lines.

Animals and other research organisms

Policy information about studies involving animals; ARRIVE guidelines recommended for reporting animal research, and Sex and Gender in Research

Laboratory animals	wild-typeC57BL/6, male, 6-10 weeks old.<br>Adipoq-Cre, male, C57BL/6J background, 6-10weeks old.<br>Ythdf1 flox/flox, male, C57BL/6J background, 6-10weeks old.<br>All mice were housed in a pathogen-free and climate-controlled environment (22–25°C, 40-60% humidity) with a 12-h light–dark cycle that provided free access to food and water unless stated otherwise.
Wild animals	The study did not involve wild animals.
Reporting on sex	Only adult male mice were used in our experiments. In this study, we find the functional role of Adipose Ythdf1 in controlling obesity and related metabolic diseases. Under HFD feeding condition, male mice were more prone to develop metabolic dysfuncion than female mice [PMID: 19136652 & 34599964]. Thus, we studied in male mice. Further studies are needed to investigate whether adipose Ythdf1 also has protective role against HFD-induced obesity in female mice.
Field-collected samples	The study did not involve field-collected samples.
Ethics oversight	All animal studies were performed in compliance with the Guide for the Care and Use of Laboratory Animals by the Medical Experimental Animal Care Commission of Zhejiang University. All animal studies used the protocol that has been approved by the Medical Experimental Animal Care Commission of Zhejiang University (ZJU20220512).

Note that full information on the approval of the study protocol must also be provided in the manuscript.
